# Influences of Immunocastration on Endocrine Parameters, Growth Performance and Carcass Quality, as Well as on Boar Taint and Penile Injuries

**DOI:** 10.3390/ani10020346

**Published:** 2020-02-21

**Authors:** Susanne Zoels, Simon Reiter, Mathias Ritzmann, Christine Weiß, Jasmin Numberger, Aneka Schütz, Peter Lindner, Volker Stefanski, Ulrike Weiler

**Affiliations:** 1 Clinic for Swine, Ludwig-Maximilians-University Munich, Sonnenstrasse 16, 85764 Oberschleissheim, Germany; simonReiter@gmx.at (S.R.); schweineklinik@med.vetmed.uni-muenchen.de (M.R.); c.weiss@med.vetmed.uni-muenchen.de (C.W.); jasmin-stark@gmx.de (J.N.); 2 Department of Safety and Quality of Meat, Max Rubner-Institut, E.-C.-Baumann-Strasse 20, 95326 Kulmbach, Germany; aneka.schuetz@mri.bund.de; 3 Bavarian State Research Center for Pig Farming Schwarzenau, Stadtschwarzacher Strasse 18, 97359 Schwarzach am Main, Germany; 4 Institute of Animal Science, Hohenheim University, Schloss Hohenheim 1, 70599 Stuttgart, Germany; volker.stefanski@uni-hohenheim.de (V.S.); weiler@uni-hohenheim.de (U.W.)

**Keywords:** immunocastration, pigs, boar taint, gonadotropin releasing hormones, GnRH antibody, carcass quality, testosterone, penile injury, androstenone, skatole

## Abstract

**Simple Summary:**

Surgical castration of male pigs is associated with pain. Improvac^®^, a GnRH vaccine induces an endogenous immune reaction that leads transiently to a decrease in testicular steroid synthesis after second vaccination. Investigating consequences of different vaccination schemes revealed that GnRH vaccination reliably prevents boar taint, if the manufacturers’ recommendations are applied. It had beneficial effects on animal welfare as it reduced penile injuries. Animals showed improved feed efficiency, leaner carcasses, and lower PUFA (polyunsaturated fatty acids) percentages than surgically castrated animals. Thus, immunocastration offers a reliable and animal friendly alternative to surgical castration.

**Abstract:**

Castration of male pigs without anesthesia is a significant welfare issue. Improvac^®^, a GnRH vaccine induces an endogenous immune response leading to a decrease in testicular steroids. Consequences of different vaccination schemes on testicular function and carcass quality were evaluated in immunocastrated boars (IC), surgical castrates (SC), and entire males (EM). Therefore, 128 male piglets were assigned to five treatment-groups and a long term follow-up group. IC groups received two vaccinations (V1, V2) with Improvac^®^ at 8 and 12, 12 and 16, or 12 and 18 weeks. Testosterone-concentrations decreased significantly two weeks after V2 in feces and dropped in serum from V2 to slaughter (S) except IC-8/12 without differing significantly. GnRH-binding results indicated the highest values for IC-12/18 animals. While IC-12/16 and IC-12/18 animals showed boar taint compounds below the threshold levels, two IC-8/12 animals had concentrations above the threshold level. Feed-efficiency was higher in EM than in SC with IC in between. In IC compared to EM, a decreasing amount of polyunsaturated-fatty-acids was obvious and GnRH-vaccination reduced penile injuries. The examined vaccination protocols reduce penile injuries, improve feed efficiency and carcass quality, and reliably prevents boar taint, if manufacturer’s recommendations concerning vaccination schedules are applied. Therefore immunocastration offers a reliable, animal friendly alternative to surgical castration.

## 1. Introduction

Castration of male piglets is a serious surgical procedure associated with pain and is thus a significant welfare issue [1]. To voluntarily end surgical castration of boars in Europe by 2018, the European Commission and representatives of all the stakeholders committed themselves into a roadmap. The designated timeline has passed, but the aim is far from being reached. In 2013, German legislation banned the procedure of surgical castration without anesthetics after 2018 but postponed this deadline up to two further years until the end of 2020. Pork production with entire male pigs has been proposed as animal friendly alternative over the last years but the main issues in product quality and behavioral problems of entire male pigs are still largely unsolved [2,3]. The active immunization of boars against endogenous gonadotropin releasing hormones (GnRH) is an effective alternative that eliminates boar taint [4] and reduces aggressive and sexual behavior [5]. Dunshea et al. [4] also described superior growth performance and leaner carcass composition in immunocastrated boars in comparison to barrows. The GnRH vaccine (Improvac^®^, Zoetis Deutschland GmbH, Berlin, Germany) consists of a truncated GnRH fragment (AS 2–10) conjugated to a large carrier protein and induces an endogenous immune reaction leading to a high level of GnRH antibodies approximately two weeks after the second vaccination [4]. The increasing antibody titer against GnRH subsequently reduces LH secretion by the pituitary gland and therefore leads to a decreasing steroid synthesis in the Leydig cells of the testes [6], concomitantly low boar taint levels [4] and reduced aggressive and sexual oriented mounting [5], respectively.

In the present study, the recommended manufacturer’s vaccination scheme, two vaccinations within an interval of at least four weeks and the second vaccination four to six weeks before slaughter (S), was deliberately modified to improve practicability, flexibility, user-friendliness, and vaccination success, especially for commercial farm use. Other studies using different vaccination schemes especially early vaccination, already exist. Andersson et al. [7] as well as Brunius et al. [8] and Einarsson et al. [9] reported the successful use of Improvac^®^ earlier than recommended with a first vaccination at ten and a second vaccination at 14 weeks of age (wk). In the present study, the effect of a first Improvac^®^ vaccination at eight and the second at twelve weeks was compared to two vaccination schemes with a later onset of the immunization effect. The consequences were monitored by measuring the GnRH binding in blood and testosterone concentrations in blood and in feces. Moreover, consequences of these vaccination schemes on carcass quality, boar taint, and penile injuries were evaluated and compared to the castrates and entire males.

## 2. Materials and Methods

The aim of the present study is to assess different vaccination schemes of a GnRH vaccine (Improvac^®^, Zoetis Deutschland GmbH, Berlin, Germany) and evaluate their influences on endocrine parameters, growth performance, and carcass quality as well as their influences on boar taint, penile injuries, and size of testis and accessory glands. Therefore, data from three groups of immunocastrated boars were collected and compared to castrates and entire males. Additionally, long term effects of Improvac^®^ were investigated in one group of immunocastrated boars.

### 2.1. Animals and Treatment

In an experimental farrow-to-finish farm in Bavaria, Germany with 230 sows, a total of 128 male piglets (Piétrain × Large White/Landrace) were randomly assigned to five treatment groups of 24 animals each (surgical castrates (SC), entire males (EM), three groups of immunocastrated boars (IC)) and additionally eight animals of a long term follow-up group (IC-12/18X) (Table 1). SC were surgically castrated without pain relieve within the first week of life according to the legal requirements. EM were fattened as entire boars. The immunocastrated animals (IC) received two vaccinations (V1, V2) with Improvac^®^ (2 mL s.c. at the base of the ear) either at an age of 8 and 12 (IC-8/12), 12 and 16 (IC-12/16), or 12 and 18 wk (IC-12/18), respectively. The animals of the long term follow-up group (IC-12/18X) designated for the evaluation of long term effects of Improvac^®^ were treated similar to IC-12/18 but slaughtered later at an age of 39 wk. Adverse tissue reactions after vaccinations were recorded.

### 2.2. Housing Conditions and Slaughtering

After a suckling period of four wk, the animals of the trial were raised until the age of ten wk and subsequently transferred to the fattening unit. In the fattening unit, all animals except IC-12/18X were kept in one building in groups of twelve animals of the same treatment group in partially slatted pens of 13 m^2^ (1.08 m^2^/pig). Thus, two pens per treatment were included into the study. Feed and water (three nipples per pen) were offered ad libitum throughout the fattening period. Composition of the feed differed according to age but not between groups (two-phase feeding: feed I: 13.86 MJ ME; 19.8% CP (crude protein) until 17 wk; feed II: 13.53 MJ ME; 17.8% CP after an age of 17 wk). IC-12/18X pigs were raised in a separate building also in a partially slatted pen but with more space allowance (1.39 m^2^/pig) until they reached approximately 120 kg. Thereafter the group was splitted and further housed in two identically sized pens (2.78 m^2^/pig). The IC-12/18x group was designated to be slaughtered if at least one animal resumed the typical boar behavior (mounting, penile extrusion) during daily routine observations, which occurred in this trial at the age of 39 wk.

Data collection to evaluate fattening performance was carried out between an average live weight of about 30 kg (30.4 ± 1.8 kg) and 120 kg (120.9 ± 7.1 kg) and comprised a period of 90.5 ± 5.8 days. Group SC, EM, IC-8/12, IC-12/16, and IC-12/18 were slaughtered in two consecutive batches (BA) (BA1: 24 wk; BA2: 26 wk) at the in-house abattoir. The average hot carcass weight (HCW) was 97.3 ± 6.6 kg. All animals of BA1 were slaughtered at the same day, whereas those of BA2 were delivered on two consecutive days to the slaughterhouse due to the low capacity of the facility and the higher number of animals in BA2.

### 2.3. Sample Collection

#### 2.3.1. Blood Sampling

Blood samples were taken from all animals during exsanguination at slaughterhouse. Serum was collected after clotting by centrifugation at 2000× *g* for 10 min and was stored at −20 °C until determination of testosterone concentration and GnRH binding. Additionally, blood samples of randomly selected animals, that were collected before second vaccination for diagnostic screening were stored for further analyses.

#### 2.3.2. Feces Sampling

Feces were collected from randomly selected marker animals every two weeks between an age of 12 wk and 22 wk (IC-12/18X: 38 wk (except wk 26)). Samples were collected during defecation or removed with gloves from the rectum carefully and stored at −20 °C until determination of testosterone concentrations.

#### 2.3.3. Adipose Tissue Sampling

From all animals, fatty acid composition and iodine values (IV) were analyzed from back fat samples (10 × 15 cm from the neck region of left carcass half) collected at S. IV values > 70 were used as an indicator of soft fat [10]. Boar taint compounds (androstenone and skatole concentration) were only measured in back fat samples (approx. 25 × 30 mm from neck region of the right carcass half) from EM and IC.

#### 2.3.4. Sampling of Penis, Testis, and Accessory Glands

From each EM and IC the penis covered with the preputial sheet were collected at the slaughter line during evisceration, where the urogenital tract was excised. The preparation and evaluation of the penis samples were performed according to Weiler et al. [11]. Consecutively, the Pars libra penis was evaluated for different types of lesions: wounds, scars, hematomas, changes of the ridge (slightly hypertrophic, slightly hypertrophic with abrasions). The size of the respective wounds and scars was recorded for each specimen according to a size-score 0.1–0.3 cm, >0.3–0.6 cm, >0.6–1 cm, >1 cm. Samples with injuries > 1 cm, with suppuration or losses of a part of penis were classified as “severe injuries” [11]. Number of wounds, scars, and hematomas add up to the total number of injuries. Testes and accessory glands were systematically evaluated only in IC groups. Additionally, samples of two boars were evaluated as an example for the EM group. Length and width of seminal vesicles, prostate gland, and bulbourethral gland as well as length, width, and weight of the right testicle was measured of each IC animal.

### 2.4. Analytical Methods

#### 2.4.1. Determination of Boar Taint Compounds

Androstenone and skatole concentrations were measured by stable isotope dilution analysis described by Fischer et al. [12]. The limit of quantification (LOQ) was 60 ng/g fat for androstenone and 1 ng/g fat for skatole [12]. For tainted carcasses the threshold limits of 1000 ng/g fat for androstenone and 200 ng/g fat for skatole, respectively, were used [4,13]. One back fat sample out of IC-12/16 was too small for laboratory analysis and therefore excluded from final evaluation.

#### 2.4.2. Determination of Fatty Acid Composition

Fatty acids were analyzed by gas chromatography (GC) using a Hewlett Packard 6890 series system with a J & W Scientific DB-23 capillary column (60 m × 0.25 mm, i.d. 0.25 μm; Agilent Technologies, Inc., Santa Clara, CA, US) and a flame ionization detector. Sample preparation was performed as described by Schulte and Weber [14]. In brief, back fat samples were homogenized and melted with butylated hydroxytoluol (BHT). For transesterification from fatty acids to methyl esters, an aliquot of the liquid fat was mixed with toluene and trimethylsulfonium hydroxide (TMSH). Then, the sample was injected into the GC system. GC conditions are as follows: injection temperature was 250 °C; carrier gas was hydrogen, 1.4 mL/min, 1:10 split and 1:50 split (for 16:0; 18:0; 18:1 cis 9; 18:1 cis 11; 18:2 cis 9,12), respectively, column temperature program, 80 °C (5 min), up to 190 °C (rate 2 °C/min), up to 220 °C (1 °C/min), 220 °C (15 min); detector temperature 250 °C. For chromatogram evaluation OpenLAB CDS ChemStation Workstation (Agilent Technologies, Inc., Santa Clara, CA, US ) was used. Calculation of fatty acids was based on the peak area of detected fatty acids (area percentage) as further described by Schwalm et al. [15]. Groups of fatty acids (saturated fatty acids: SFA, monounsaturated fatty acids: MUFA, polyunsaturated fatty acids: PUFA) were calculated based on all analyzed fatty acids.

#### 2.4.3. Quantification of GnRH Binding in Serum Samples

Success of vaccination was quantified by measuring GnRH binding in serum. Binding was determined with an in-house assay, based on 125I-GnRH. GnRH-iodination was carried out with the solid phase Iodogen-method according to Salacincki et al. 1981, using 1 µg Iodogen/cup, 200 µCi 125I (Na125I, Hartmann Analytik GmbH, Braunschweig, Germany, I-RB-31.) and 200 ng GnRH (PEP-168, Fisher Scientific GmbH, Schwerte, Germany) diluted in 0.5 M phosphate buffer (pH 7.4). After an incubation period of 3 min, the free iodine was separated from the iodinated peptide with an anion exchange resin column. The specific activity was about 200 nCi/ng GnRH. For determination of the GnRH binding 15,000 cpm 125I-GnRH (corresponding to 17.5 pg GnRH, “total counts”) in 100 µL in 0.1 M phosphate buffer were incubated with 5 µL of serum and 200 µL in 0.1 M phosphate buffer with the addition of BSA (0.1%) at 4 °C for 24 h. Thereafter, bound free separation was carried out with dextran-coated charcoal (0.5%) in 1 mL H_2_O and subsequent centrifugation. The supernatant was counted for one minute in a gamma counter. As controls, a pool sample of IC with a good response (pool A) and a pool sample of non-vaccinated EM (pool B) were measured within each assay. The binding percentage (%) of the biological samples was calculated (total counts = 100%). The binding of pool A was 54.0 + 5.37%; (CV: 10%), the non-specific binding determined with pool B was 4.5 + 1.27%, (CV: 28%; range 5.81% to 2.36%).

#### 2.4.4. Determination of Testosterone Concentrations in Serum

Testosterone concentrations in serum were determined in duplicate with a direct in-house radioimmunoassay (RIA). In brief, 20 µL serum was incubated with ^3^H-testosterone and antiserum. The antiserum had been raised in a rabbit against testosterone-3CMO-BSA and was used at a final dilution of 1:144,000. Cross reactivity was 67% with 5αDHT, and below 2% for other tested steroids. Charcoal-treated serum (20 µL) was added to the calibration curve to compensate for substrate effects in case of measurements in serum. Bound free separation was carried out with 0.5 mL ice cold solution of dextran-coated charcoal (0.5%) in H_2_O and subsequent centrifugation. The supernatant was transferred into counting vials with scintillation fluid and counted in a beta-counter. Intra-assay and inter-assay variability was determined with pig serum samples and were below 8% each. Precision was determined with samples of spiked pig serum. The mean recovery rate of added concentrations was 110%.

#### 2.4.5. Determination of Testosterone Concentrations in Feces

The radioimmunological analysis of testosterone concentrations in fecal samples was carried out after extraction according to Wesoly et al. [16]. Extraction comprised a two-step solvent distribution. In brief, fecal samples of about 0.5 g each were dissolved in 500 μL of water, then 4 mL methanol was added. After mixing the sample for 30 min, 3 mL petroleum ether was added. After mixing and centrifugation, the petroleum ether was discharged. An aliquot of 100 μL of the remaining methanol/water fraction was further diluted with 600 µL water and extracted with 5 mL of 7:3 (v/v) petroleum ether/ethyl acetate. After incubation for 30 min and freezing, the supernatant was collected and evaporated. The residue was reconstituted with 100 µL phosphate buffer and the hormone concentrations determined radioimmunologically as similarly described above for measurements in serum. The average recovery rate for ^3^H-testosterone from fecal samples was 50.2%. Intra-assay and inter-assay variability in fecal samples were below 5% each. Precision was determined with spiked fecal samples and revealed a mean recovery rate of 76.8% [16].

### 2.5. Statistical Analysis

All statistical analyses were performed in IBM SPSS Statistics Program (Version 23). All data were tested for normal distribution by Shapiro-Wilk test. In normally distributed parameters, differences between trial groups were tested with ANOVA (Univariate ANOVA, fixed effects: group and batch) and Bonferroni as post-hoc test. A repeated measure analysis (GLM) was carried out to evaluate the effects of time and group × time on fecal testosterone concentration. To compare normally distributed parameters within a group the paired samples test (*t*-Test) was used. As the number of scars and wounds were not normally distributed, the nonparametric Kruskall-Wallis test, followed by Mann-Whitney U test and Bonferroni-Holm correction was used for analysis of differences between groups. For the influence of group and batch on androstenone and skatole concentration data were transformed to ranks and subsequently ANOVA was performed [17]. The relationship between GnRH binding and testosterone concentration at S; androstenone and skatole concentration; and androstenone/skatole concentration and testosterone concentration at S was analyzed by calculating the Pearson correlation. For regression analysis between GnRH binding and serum testosterone concentration the Pearson correlation coefficient was used. Associations of categorical variables/percent values with group membership were analyzed using the chi-square test. *p*-values of <0.05 were considered as statistically significant.

## 3. Results

Data from 23 SC, 24 EM, and 79 IC animals (total: *n* = 126) were statistically analyzed. Two animals (one in group SC and IC-12/18x, resp.) died during the trial and were excluded from the final evaluation. In IC groups, no adverse tissue reactions could be observed in any animal after vaccination.

### 3.1. Fecal Testosterone Concentrations

Samples of five animals of SC, EM, IC-8/12, IC-12/16, six animals of IC-12/18 and seven samples in case of IC-12/18X, respectively were analyzed for fecal testosterone concentrations every two weeks. Analysis by GLM repeated measures revealed no significant effect of time (df = 1.34, F = 1.85, *p* = 0.180) but a significant effect of time × group (df = 6.98, F = 3.32, *p* = 0.008) on fecal testosterone concentrations (Table 2).

**Figure 1:**
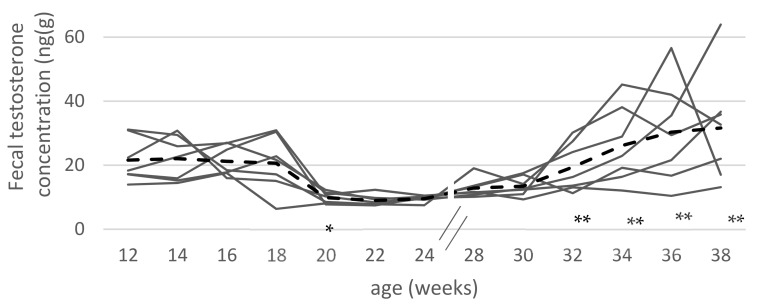
Individual (-) and mean (- -) fecal testosterone concentrations (ng/g) of animals in the long term follow-up group IC-12/18X (*n* = 7) from 12th to 38th week of age; * indicate significant (*p* < 0.05) differences compared to two weeks earlier; ** indicate significant difference to testosterone concentration compared to week 30.

After V2, a significant decrease of fecal testosterone concentration to those levels of SC was measured (*p* < 0.05) in all IC groups. IC-12/18X animals showed increasing testosterone concentrations at the end of the investigation period (30th to 38th wk). The testosterone concentrations at the end of the study were similar to values obtained in EM at 22nd wk (Figure 1).

### 3.2. GnRH Binding and Serum Testosterone Concentrations

**Figure 2:**
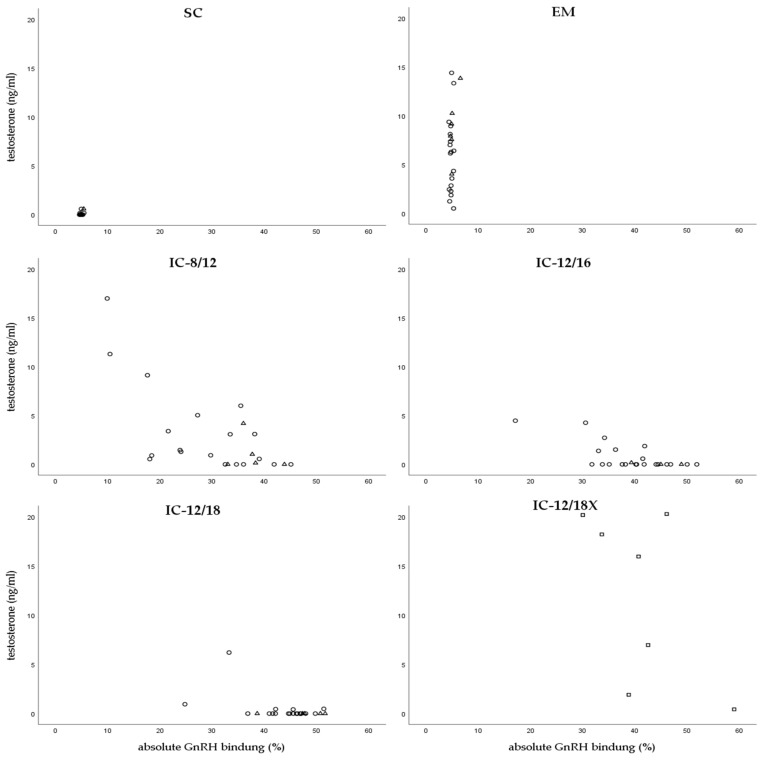
Testosterone levels (ng/mL) and absolute GnRH binding (%) in serum of animals slaughtered at batch 1 (∆), batch 2 (o), and batch 3 (□).

GnRH binding and testosterone levels were analyzed in serum of all animals at time of S (Table 3 and Figure 2). Additionally, serum of the animals sampled for diagnostic screening at V2 were analyzed. At S, SC revealed only basal testosterone levels whereas EM had significantly higher testosterone levels with a higher variability (min: 0.51 ng/mL; max: 14.39 ng/mL). In these two non-vaccinated groups, all GnRH binding values were below cutoff for nonspecific binding. GnRH binding at S was significantly influenced by both, group and batch (group: *p* < 0.001; batch: *p* < 0.05), whereas testosterone concentration in serum was only influenced by group (*p* < 0.001). In IC groups (except IC-12/18X) testosterone concentration correlated with GnRH binding in serum at S (r = −0.68; *p* < 0.001; *n* = 72) and regression analysis revealed a significant effect of GnRH binding on testosterone concentrations (B = −0.196, SE(B) = 0.026, *p* < 0.001).

At V2, neither group nor batch had a significant effect on testosterone level or absolute GnRH binding. At this time however, IC-8/12 tended to have lower testosterone levels than IC-12/16 and IC-12/18 (0.90 ± 0.62 ng/g vs. 3.22 ± 1.56 ng/g and 2.42 ± 1.33 ng/g) and absolute GnRH binding varied in IC groups between 16.79 ± 7.00% (IC-8/12) and 24.58 ± 3.36% (IC-12/18). In IC-12/16 and IC-12/18 testosterone concentration in serum decreased significantly from V2 to S (*p* < 0.05).

### 3.3. Fattening Performance and Carcass Quality

Parameters of fattening performance as well as fat quality characteristics are summarized for all groups slaughtered at 120 kg in Table 4. Hot carcass weight ranged between 94.4 and 98.5 kg without differing significantly between groups. Concordantly, average daily weight gain (ADW) did not differ significantly between groups, although batch had a significant effect (*p* < 0.001). In contrast, Feed conversion ratio (FCR) for gain between 30 and 120 kg live weight varied significantly between groups (*p* < 0.001) with lowest feed/gain values in EM and the highest amount of feed needed for IC-8/12.

EM revealed lowest and SC highest percentage of IMF with IC values intermediate. Only group had a significant effect on IMF (*p* < 0.001) in contrast to batch. With regard to fatty acid composition (PUFA, MUFA, SFA), group and batch had a significant effect (group: *p* < 0.001, batch: *p* < 0.05), except PUFA values, where only group seemed to have a significant effect (group: *p* < 0.001, batch: ns). EM had the highest percentage of PUFA and the lowest of SFA and MUFA compared to SC and IC groups, whereas IC displayed lower PUFA values compared to SC, with IC-12/16 indicating the lowest value. 83.3% of EM had IV values over 70 compared to SC (43.5%) and IC groups (16.7%).

### 3.4. Levels of Boar Taint Compounds

**Figure 3:**
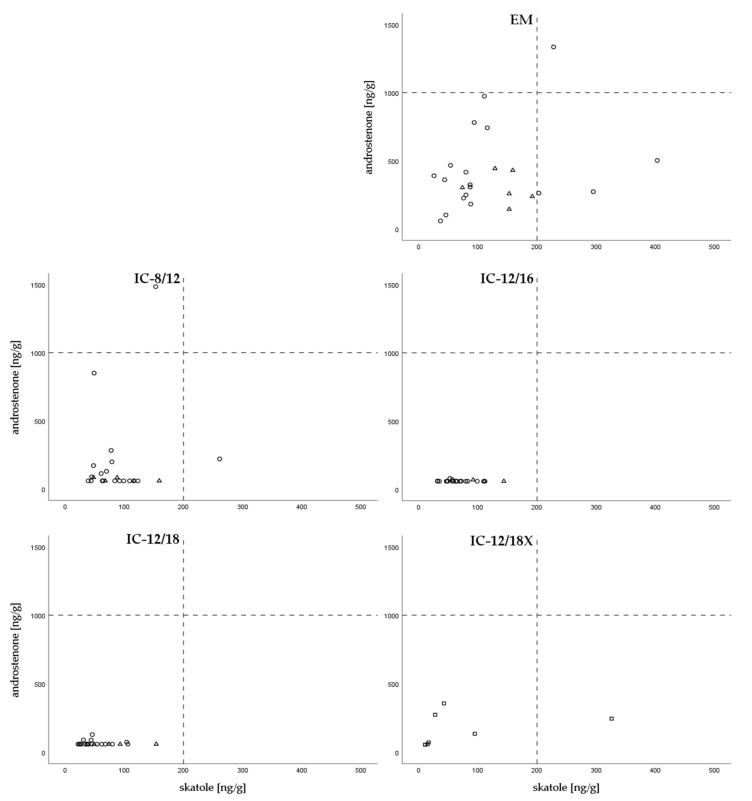
Androstenone (ng/g) and skatole (ng/g) levels of animals slaughtered at batch 1 (∆), batch 2 (o), and batch 3 (□) with threshold levels for androstenone (1000 ng/g) and skatole (250 ng/g) of group EM, IC-8/12, IC-12/16, IC-12/18, and IC-12/18X.

Androstenone and skatole concentrations in back fat were analyzed in samples from 78 IC and 24 EM. Both, androstenone and skatole concentrations were significantly affected by group (*p* < 0.001). Moreover, batch had a significant effect on skatole concentration (*p* < 0.001) but not on androstenone levels. No significant correlation between androstenone and skatole was found in EM (r = 0.228; *p* > 0.05, *n* = 24) or in IC groups (r = 0.220; *p* > 0.05, *n* = 71). However, androstenone concentrations in adipose tissue and testosterone concentrations in serum at S were significantly correlated (r = 0.570; *p* < 0.001, *n* = 78) in IC animals. EM revealed significantly higher androstenone concentrations in adipose tissue (407.8 ± 291.6 ng/g) than IC groups (111.6 ± 188.8 ng/g, *p* < 0.001) and significantly higher skatole concentrations (125.6 ± 88.4 ng/g) than IC groups except from IC-8/12 (skatole 73.0 ± 49.5 ng/g; *p* < 0.05). The lowest average concentrations of androstenone and skatole were measured in IC-12/16 (androstenone: 62.0 ± 16.2 ng/g, skatole: 71.0 ± 29.0 ng/g) and IC-12/18 (androstenone: 66.1 ± 16.3 ng/g; skatole: 57.0 ± 32.2 ng/g). The relationship between androstenone and skatole concentrations in adipose tissue of EM and IC groups are given in Figure 3. Androstenone concentrations in fat tissue was significantly correlated to PUFA (r = 0.487; *p* < 0.001, *n* = 71) and SFA (r = −0.351; *p* = 0.003; *n* = 71) in IC groups but not in EM group (PUFA: r = −0.118; *p* > 0.05; SFA: r = −0.055; *p* > 0.05; *n* = 24).

### 3.5. Penile Injuries

Total of 103 animals were evaluated for penile injuries (Table 5). 91.7% of EM had signs of injuries (scars, wounds, hematomas). In ICs, this percentage ranged between 16.7% (IC-8/12) and 41.7% (IC-12/18). IC-12/18X had more injuries than any other IC group (71.4%). However, these differences were significant between IC-8/12 and IC-12/18.

Total of 20.8% of EM had slightly hypertrophic ridges and 12.5% showed hypertrophic ridges with lesions. This was generally lower in IC, irrespectively of the treatment group; no ridge alteration in IC-12/18X and IC-12/18 could be detected, whereas 4.2% of IC-8/12 showed slightly hypertrophic ridges and 8.3% hypertrophic ridges with lesions. In IC-12/16 4.2% showed slightly hypertrophic ridges, however, no animal showed a hypertrophic ridge with lesions. The amount of animals with hypertrophic ridges differed significantly within groups (*p* = 0.009).

### 3.6. Accessory Glands

Length of testes and accessory glands (Gl. bulbourethralis, Gl. vesicularis, Gl. prostatica) were evaluated in 66 IC animals but only in 2 EM (Table 6). Because of unbalanced sampling, the effect of batch could not be evaluated for these parameters. At S, size and weight of testes and accessory glands correlated significantly to testosterone and androstenone concentrations. For the statistical analysis, groups IC-12/18X and EM were not included in the group comparison because of the low sample sizes in these groups.

## 4. Discussion

It is well-known that surgical castration without anesthesia is a painful procedure [1,18,19,20,21] and numerous investigations concerning alternatives, like GnRH vaccination or boar fattening already exist [22,23,24]. However, there are open questions yet to be answered. This study gives insights into imunocastrated boars (IC) compared to EM and SC and deals with modified vaccination times and their consequences on endocrine parameters as well as on carcass quality, boar taint, penile injuries, and behavioral changes.

Concordantly to Dunshea et al. [4], fecal testosterone concentrations decreased significantly from V2 to V2 + 2 wk and testosterone concentrations in serum from V2 to S in all IC groups independently of vaccination scheme except animals from group IC-8/12. These results are supported by results concerning behavioral observations of other authors [5,25] showing behavioral changes two weeks after the second vaccination against GnRH [4,25]. The testosterone concentrations of IC-8/12 may be explained by the early pubertal stage of this group at the first sampling in the 12th wk and reflected by lower testosterone concentrations at V2 than in all other groups. Recovery of fecal testosterone concentration in IC-12/18X animals in the 32th wk, 10 weeks after V2, demonstrates that the effect of GnRH vaccination is reversible. The duration between second vaccination and the resumption of testicular function varies between studies. Zamaratskaia et al. [26] reported inhibitory effects of vaccination against GnRH on male behavior and boar odor that can persist for 22 weeks after V2. In contrast, Claus et al. [27] and Rottner and Claus [28] described individual differences in the resumption of testicular steroidogenesis from 10 to 24 weeks after V2. In the present study, fecal testosterone concentration began to increase at least ten weeks after V2.

At S, no significant differences in serum testosterone concentration could be detected between IC groups (except IC-12/18X). These results are in accordance with Einarsson et al. [9] showing no differences between early and standard vaccinated IC groups on the day prior to S. The androgen production in adrenal cortex, beside the testis as main localization of androgen formation [29], explains the detected basal testosterone concentrations in SC and successfully vaccinated IC at S in this study. In consistence with Einarsson et al. [9] and Dunshea et al. [4] the high variability in testosterone concentration of EM at S is due to different stages of pubertal development and effects of social ranking on testicular function [30,31].

For the GnRH antibody increase after V2, individual differences seem to play an important role [4,28,32,33]. Rottner and Claus [28] described maximum GnRH antibody peaks eight to eleven days after V2, whereas Claus et al. [33] reported maxima already four to six days after V2. After this peak, GnRH antibody titers start to decrease continuously, explaining lower binding capacity in the early vaccinated groups in comparison to standard vaccinated groups [8]. The GnRH binding results from the present study indicate the highest values for IC-12/18 animals at time of S because of the shortest interval between V2 and S compared to other groups. The increasing GnRH binding at V2 from IC-8/12 to IC-12/18 should not be overrated as differences between IC-12/18 and IC-12/18X could be measured despite the same vaccination schedule.

Various studies about the efficacy of Improvac^®^ in preventing boar taint support the conclusion that the GnRH vaccine unequivocally reduces both androstenone and skatole [4,26,34]. The close relationship between androstenone concentration and testis function explains why suppressed testis function leads to lower androstenone concentrations [30,35]. The changes in skatole concentrations are the consequence of the inhibitory effect of androstenone on the expression of CYP2E1 [36], another important key enzyme in skatole metabolism beside CYP2A [36,37]. Thus, high androstenone concentrations also tend to lead to high skatole concentrations [38]. As commonly accepted, threshold values of 1000 ng/g fat for androstenone and 200 ng/g fat for skatole were used in this investigation [4,13]. Indeed, IC also displayed lower average androstenone and skatole concentrations compared to EM. However, differences between vaccination groups were noticeable. While 100% of IC-12/16 and IC-12/18 animals showed values below threshold levels, two animals of IC-8/12 had concentrations above, probably due to the long time interval from the second vaccination to S (V2 to BA2, 14 weeks).

In this study, ADW tended to be higher and FCR and IMF as carcass quality parameter tended to be lower in EM than in SC with IC in between. Reduced steroid production after the second vaccination could be an explanation for the intermediate IMF content of IC compared to EM [39,40]. Thus, IC start to eat more, grow faster [4,26,41], and show less social and sexual activity [5] providing the large amount of energy supply for fat deposition that results in higher IMF values. These results are confirmed by several studies [4,5,27,41,42] and demonstrate the mechanism of Improvac^®^ preventing the stimulation of Leydig cells and therefore the secretion of testicular hormones by binding GnRH because of endogenous antibodies.

Moreover, vaccination affected fat quality characteristics and fatty acid composition. As in this study, former studies concerning fat quality characteristics reported boars having lower amounts of saturated and higher amounts of unsaturated fatty acids than females or castrates [10,42,43,44]. Fat quality parameters of IC groups differed from EM group and corresponded close to fat quality characteristics of SC animals. Pauly et al. [42] and Sauer [43] reported decreasing degrees of saturation in adipose tissue from SC to IC and from IC to EM. The high level of unsaturated fatty acids in boars is mainly caused by the higher level of linoleic (18:2) and linolenic (18:3) acids and lower level of palmitic acid (16:0).

Whereas a decreasing amount of PUFA in IC with increasing fat deposition was obvious, the results of SC are astonishing, as the higher amount of IMF was not accompanied by a higher percentage of SFA. The iodine values support these differences for these groups, as all IC groups had lower iodine values than SC. Even if fatty acid composition is also affected by other factors than gender, breed, feed composition, and age [45,46,47], these factors could be excluded because of standardized conditions for all animals in the present study.

In a previous publication from our group, the occurrence of penile injuries in intensive production systems as well as in wild living boars has been discussed in detail [11]. In a recently published study we could further show that immunocastration reduces the frequency and severity of penile injuries compared to EM of same age and weight [48]. More frequent occurrence of sexually oriented mounting behavior in EM is discussed as a reason for the higher incidence of penile injuries compared to IC [11,48,49], because the extrusion of the penis during sexually oriented mounting enables other pen mates to bite and vulnerate the penis [11,48]. The results from the present study agree with those of Reiter et al. [48] and explain the significantly lower rate of penile injuries in IC compared to EM as a consequence of decreasing sexual behavior. Moreover, differences between the IC groups show that the timing of the second vaccination has a tremendous effect on the amount of animals with such injuries. Earlier V2 corresponded clearly to a lower number of scars per animal and a reduced percentage of animals with lesions and severe injuries. These results are consistent with the behavioral results of Rydhmer et al. [5], who demonstrated that immunocastration significantly reduces aggressive and sexual behavior in male pigs after the second vaccination but had no significant effect compared to entire males before second vaccination. The increasing number of penile injuries in IC-12/18X is explained by the long time interval from V2 to S (21 weeks). As indicated by the increasing fecal testosterone levels during the last eight weeks of the fattening in this group it is likely that the resumption of male behavior with sexual oriented mounting occurred.

Moreover, size of accessory glands is influenced significantly by testicular function in the present study as indicated by a good correlation between size of accessory glands or testes weight and testosterone concentration in serum or androstenone concentration in fat tissue. Concordantly, the vaccination schedule influenced the size of accessory glands, which is in accordance with a number of other studies dealing with immunocastration [8,9,50,51]. However, only the bulbourethral and vesicular glands showed significant differences between IC groups in the present study. In contrast to some of those studies, the reduction in size was especially large for earlier vaccinated animals. As measured by bulbourethral and vesicular glands, the greatest immunocastration effect was in group IC-12/16, possibly because of the longer duration between V2 and S compared to IC-12/18. In IC-8/12, it might be that the resumption of testes function of some individuals had already started at S, twelve to 14 weeks after V2. Rottner [52] descripted some immunocastrated pigs with an early resumption of Leydig cell function had lower testosterone levels and lighter accessory glands than other study animals that showed later resumption of Leydig cell function. A comparison to EM or IC-12/18X could not be drawn because of the low sample size of accessory glands but size of bulbourethral and vesicular glands and testes in these two groups were in the same range and tended to be higher than in IC.

## 5. Conclusions

It can be concluded that GnRH vaccination reliably prevents boar taint, if the manufacturer’s recommendations concerning vaccination schedules are applied. The early vaccination protocol used in the present study cannot be recommended with regard to a reliable suppression of boar taint. Fecal testosterone measurement provides a successful method to evaluate vaccination success without stressful blood sampling. GnRH vaccinations have beneficial effects on animal welfare in pork production systems as it influence behavior and thus the frequency and severity of penile injuries. Additionally, IC had improved feed efficiency compared to SC, except the early vaccinated IC group, leaner carcasses and even lower PUFA percentages than SC. Thus, immunocastration offers a reliable and animal friendly alternative to surgical castration.

## Figures and Tables

**Table 1:** Treatment groups, age and weight (kg; mean± SD) at vaccination (V1, V2) and age at slaughter (wk).

Group	*n*	Age (wk) at Vaccination		Weight (kg; Mean ± SD) at Vaccination		Number of Animals Slaughtered at an Age of		
		V1	V2	V1	V2	24 wk	26 wk	39 wk
SC	24	--	--	--	--	4	19	--
EM	24	--	--	--	--	6	18	--
IC-8/12	24	8	12	17.2 ± 2.1	31.3 ± 4.2	5	19	--
IC-12/16	24	12	16	31.9 ± 2.8	53.9 ± 5.0	3	21	--
IC-12/18	24	12	18	30.4 ± 3.2	65.3 ± 6.8	5	19	--
IC-12/18X	8	12	18	nd	nd	--	--	7

nd: not determined, wk: age in weeks, V1: first vaccination, V2: second vaccination.

**Table 2:** Fecal testosterone concentration (ng/mL) (mean ± SD) within groups SC, EM IC-8/12, IC-12/16, and IC-12/18 from 12th to 22nd week of age.

Groups	*n*	Weeks of Age					
		12th	14th	16th	18th	20th	22nd
SC	5	11.0 ± 1.6 ^a^	12.2 ± 2.9 ^ac^	10.2 ± 1.3 ^a^	10.5 ± 1.7 ^a^	11.6 ± 1.9 ^a^	10.8 ± 1.5 ^a^
EM	5	20.2 ± 17.6	26.2 ± 5.2 ^b^	34.7 ± 8.4 ^b^	31.5 ± 6.1 ^b^	34.4 ± 5.3 ^b^	37.2 ± 17.3 ^b^
IC-8/12	5	15.0 ± 2.6	8.3 ± 1.4 ^a^	10.6 ± 1.7 ^ac^	9.1 ± 2.4 ^a^	8.6 ± 2.0 ^a^	9.3 ± 1.8 ^a^
IC-12/16	5	20.3 ± 3.0 ^b^	20.0 ± 7.6 ^cb^	27.3 ± 9.3 ^d^	12.3 ± 2.4 ^a^	8.1 ± 1.3 ^a^	7.6 ± 2.0 ^a^
IC-12/18	6	18.3 ± 5.8	19.2 ± 4.2 ^c^	21.7 ± 6.5 ^ad^	31.9 ± 8.9 ^b^	10.1 ± 2.4 ^a^	10.4 ± 2.3 ^a^

Differing superscripts within a column indicate significant (*p* < 0.05) differences between groups. Surgical castrates (SC); entire males (EM); immunocastrated boars in 8th and 12th wk (week of live) (IC8/12); 12th and 16th wk (IC12/16); 12th and 18th wk (IC12/18).

**Table 3:** Testosterone concentration (ng/mL) and absolute GnRH binding (%) (mean ± SD) in serum at second vaccination (V2) and slaughter (S) within the different groups (SC, EM, IC-8/12, IC-12/16, IC-12/18, IC-12/18X).

Group	Second Vaccination			Slaughter		
	*n*	Testosterone (ng/mL)	Absolute GnRH Binding (%)	*n*	Testosterone (ng/mL)	Absolute GnRH Binding (%)
SC	-	nd	nd	23	0.06 ± 0.17 ^a^	4.85 ± 0.24 ^a^
EM	-	nd	nd	24	6.63 ± 3.94 ^b^	4.88 ± 0.44 ^a^
IC-8/12	5	0.90 ± 0.62	16.79 ± 7.00	24	2.88 ± 4.26 ^a^	30.24 ± 10.13 ^b^
IC-12/16	5	3.22 ± 1.56 ^a^	20.31 ± 8.91	24	0.71 ± 1.34 ^b^	39.58 ± 7.58 ^c^
IC-12/18	6	2.42 ± 1.33 ^a^	24.58 ± 3.36	24	0.35 ± 1.27 ^ab^	44.20 ± 6.12 ^c^
IC-12/18X	7	5.21 ± 3.57	17.51 ± 9.09	7	11.98 ± 8.66 ^c^	41.55 ± 9.41 ^c^

Differing superscripts within a column or a row indicate significant (*p* < 0.05) differences between groups. Surgical castrates (SC); entire males (EM); immunocastrated boars in 8th and 12th wk (week of live) (IC8/12); 12th and 16th wk (IC12/16); 12th and 18th wk (IC12/18) and long term follow-up group (IC-12/18X); nd: not determined.

**Table 4:** Hot carcass weight (HCW, kg), average daily weight gain (ADW; g/d), feed conversion rate (FCR, kg feed/kg gain), intramuscular fat (IMF; %), and fatty acid composition of back fat (SFA, MUFA, PUFA; %) (mean ± SD) of group SC, EM, IC8/12, IC12/16, IC12/18, and IC12/18X.

Group Parameter	SC (*n* = 23)	EM (*n* = 24)	IC-8/12 (*n* = 24)	IC-12/16 (*n* = 24)	IC-12/18 (*n* = 24)	*p*-Value
Performance parameters						
HCW (kg)	94.4 ± 6.3	97.7 ± 4.9	97.4 ± 7.4	98.5 ± 6.4	98,3 ± 7.6	n.s.
ADW (g/d)	982.5 ± 69.1	1002.1 ± 83.6	1001.9 ± 73.9	989.2 ± 54.1	1028.8 ± 98.1	n.s.
FCR (kg/kg)	2.3 ± 0.1 ^a^	2.0 ± 0.1 ^b^	2.7 ± 0.4 ^c^	2.2 ± 0.1 ^a^	2.1 ± 0.1 ^ab^	<0.001
Fat quality						
IMF (%)	1.54 ± 0.33 ^a^	1.07 ± 0.29 ^b^	1.46 ± 0.31 ^a^	1.35 ± 0.46 ^ab^	1.53 ± 0.46 ^a^	<0.001
SFA (%)	39.0 ± 1.7 ^ab^	37.9 ± 1.6 ^b^	39.9 ± 1.9 ^ca^	41.7 ± 2.5 ^d^	41.8 ± 2.1 ^d^	<0.001
MUFA (%)	44.4 ± 1.7 ^a^	41.8 ± 2.3 ^b^	43.7 ± 1.6 ^ac^	43.2 ± 1.4 ^ac^	42.8 ± 1.2 ^bc^	<0.001
PUFA (%)	16.6 ± 1.7 ^a^	20.4 ± 2.6 ^b^	16.4 ± 2.0 ^a^	15.2 ± 2.6 ^a^	15.5 ± 1.7 ^a^	<0.001
IV	69.0 ± 2.6 ^a^	73.4 ± 3.2 ^b^	68.1 ± 3.1 ^ac^	65.4 ± 4.4 ^c^	65.5 ± 3.2 ^c^	<0.001

Values with differing subscripts within a line differ significantly (*p* < 0.05). nd: not determined; IMF: % Intramuscular fat; SFA: % Saturated fatty acids of all fatty acids (100%); MUFA: % Monounsaturated fatty acids of all fatty acids (100%); PUFA: % Polyunsaturated fatty acids of all fatty acids (100%); IV: iodine value; surgical castrates (SC); entire males (EM); immunocastrated boars in 8th and 12th wk (week of live) (IC8/12); 12th and 16th wk (IC12/16); 12th and 18th wk (IC12/18).

**Table 5:** Number of scars and fresh wounds per animal (mean ± SD) and percentage of animals with lesions and severe injuries (%) of group EM, IC-8/12, IC-12/16, IC-12/18, IC-12/18X.

Group	*n*	Scars/Animal	Wounds/Animal	Animals with Lesions (%)	Animals with Severe Injuries (%)
EM	24	7.92 ± 4.75^a^	1.63 ± 1.69^a^	91.67^a^	12.5^a^
IC-8/12	24	0.17 ± 0.64^b^	0.08 ± 0.28^b^	16.67^b^	0.00^a^
IC-12/16	24	0.54 ± 0.93^cb^	0.00 ± 0.00^cb^	29.17^bc^	0.00^a^
IC-12/18	24	0.83 ± 1.17^dc^	0.08 ± 0.41^b^	41.67^bc^	4.17^a^
IC-12/18X	7	3.29 ± 3.15^ad^	0.71 ± 1.25^a^	71.43^bc^	0.00^a^

Values with differing superscripts within a column differ significantly (*p* < 0.05); entire males (EM); immunocastrated boars in 8th and 12th wk (week of live) (IC8/12); 12th and 16th wk (IC12/16); 12th and 18th wk (IC12/18) and long term follow-up group (IC-12/18X).

**Table 6:** Size (cm) of accessory glands and testes and weight (g) of testes (mean ± SD) of group EM, IC-8/12, IC-12/16, IC-12/18, IC-12/18X, and correlation between sizes and testosterone concentration in serum and androstenone concentration in fat tissue at slaughter of IC groups.

Parameter		Group (*n* =)						r Spearman Rho (*n* =)	
		IC-8/12 (21)	IC-12/16 (21)	IC-12/18 (18)	(*p*)	IC-12/18X (6)	EM (2)	Testost.	Androst.
Weight (g)		128.3 ± 105.2	58.1 ± 26.2	68.6 ± 32.6	0.128	304.2 ± 169.2	/	0.626 * (65)	0.387 * (64)
Testes (cm)	wi	4.4 ± 1.8	3.5 ± 0.9	3.8 ± 0.8	0.333	6.5 ± 2.0	5.0/7.5	0.550 * (67)	0.379 * (66)
	le	6.5 ± 1.7	5.2 ± 0.86	5.8 ± 1.1 ~	0.210	10.3 ± 3.0	10.3/8.1	0.566 * (67)	0.435 * (66)
GlBu (cm)	wi	2.1 ± 0.9^a^	1.4 ± 0.6^b^	1.6 ± 0.5^ab^	0.039	2.8 ± 1.0	7.5/4.9	0.638 * (67)	0.451 * (66)
	le	8.3 ± 2.5^a^	6.4 ± 1.2^b^	7.2 ± 1.3^ab^	0.017	11.0 ± 3.5	12.2/8.9	0.584 * (68)	0.478 * (67)
GlVe (cm)	wi	3.2 ± 2.0^a^	1.7 ± 0.7^b^	2.2 ± 0.9^ab^	0.041	5.0 ± 2.8	5.0/3.1	0.561 * (68)	0.442 * (67)
	le	4.9 ± 2.6	3.4 ± 0.9	3.8 ± 1.0	0.079	7.3 ± 3.1	11.3/7.9	0.554 * (68)	0.396 * (67)
GlPr (cm)	wi	1.0 ± 0.8	0.7 ± 0.4	0.9 ± 0.4	0.213	2.2 ± 1.0	6.2/6.9	0.486 * (68)	0.265 * (67)
	le	2.5 ± 0.8	2.2 ± 1.0	2.3 ± 0.7	0.440	4.0 ± 1.1	3.1/3.3	0.534 * (68)	0.362 * (67)

Values with differing superscripts within a column differ significantly (*p* < 0.05) between groups; ~ *n* = 17; r: correlation coefficient (Spearman Rho); * significant correlation (*p* < 0.05); wi: width; le: length; GlBu: Glandula bulbourethralis; GlVe: Glandula vesicularis; GlPr: Glandula prostatica; Testost.: testosterone in serum; Androst.: Androstenone in fat tissue; entire males (EM); immunocastrated boars in 8th and 12th wk (week of live) (IC8/12); 12th and 16th wk (IC12/16); 12th and 18th wk (IC12/18), and long term follow-up group (IC-12/18X).
